# Contribution of Dietary Supplements to Nutritional Adequacy in Various Adult Age Groups

**DOI:** 10.3390/nu9121325

**Published:** 2017-12-06

**Authors:** Jeffrey B. Blumberg, Balz Frei, Victor L. Fulgoni, Connie M. Weaver, Steven H. Zeisel

**Affiliations:** 1 Antioxidants Research Laboratory, Jean Mayer USDA Human Nutrition Research Center on Aging, and the Friedman School of Nutrition Science and Policy, Tufts University, Boston, MA 02111, USA; jeffrey.blumberg@tufts.edu; 2 Linus Pauling Institute and Department of Biochemistry & Biophysics, Oregon State University, Corvallis, OR 97331, USA; balz.frei@oregonstate.edu; 3 Nutrition Impact, LLC, Battle Creek, MI 49014, USA; 4 Department of Nutrition Science, Purdue University, West Lafayette, IN 47907, USA; weavercm@purdue.edu; 5 Nutrition Research Institute, Department of Nutrition, University of North Carolina, Kannapolis, NC 28081, USA; steven_zeisel@unc.edu

**Keywords:** vitamin/mineral supplement, NHANES, micronutrients, adults, older adults

## Abstract

Many Americans have inadequate intakes of several nutrients. The Dietary Guidelines for Americans 2015–2020 specifically identified vitamins A, C, D and E, calcium, magnesium, iron, potassium, choline and fiber as “underconsumed nutrients”. Based on nationally representative data in 10,698 adults from National Health and Nutrition Examination Surveys (NHANES), 2009–2012, assessments were made of age-group differences in the impact of dietary supplements on nutrient intake and inadequacies. Compared to food alone, use of any dietary supplement plus food was associated with significantly (*p* < 0.01) higher intakes of 15–16 of 19 nutrients examined in all age groups; and significantly reduced rates of inadequacy for 10/17, 8/17 and 6/17 nutrients examined among individuals age ≥71, 51–70 and 19–50 years, respectively. Compared to the other age groups, older adults (≥71 years) had lower rates of inadequacy for iron and vitamins A, C, D and E, but higher rates for calcium. An increased prevalence of intakes above the Tolerable Upper Intake Level was seen for 8–9 of 13 nutrients, but were mostly less than 5% of the population. In conclusion, dietary supplement use is associated with increased micronutrient intake, decreased inadequacies, and slight increases in prevalence above the UL, with greater benefits seen among older adults.

## 1. Introduction

Despite continued public health recommendations including recent dietary guidelines [1] providing guidance on healthful dietary patterns, many Americans do not adhere to these recommendations [2]. Studies have shown that Americans have inadequate intakes of several essential nutrients [3,4,5,6], despite an abundant supply of nutrient-dense foods such as whole grains, fruits, vegetables, low-fat dairy products, and lean meats [7]. Adequate intake of micronutrients is critical for health, growth and development; healthy aging; and well-being across the lifespan. The Dietary Guidelines for Americans 2015–2020 (DGA) identified vitamin A, vitamin C, vitamin D, vitamin E, choline, calcium, iron (for certain age/gender groups), magnesium, potassium and fiber as “underconsumed nutrients”; and vitamin D, calcium, potassium and fiber as “nutrients of public health concern” because low intakes are associated with a risk for chronic disease [1].

The Second National Report on Biochemical Indicators of Diet and Nutrition in the U.S. Population reported that nutrition deficiencies varied by age, gender, or race/ethnicity and could be as high as nearly one third of certain population groups; the highest levels of deficiencies were noted for vitamin B_6_, vitamin D, and iron [8]. DGA recommends consuming nutrient dense foods as part of a healthy eating pattern and, in some cases, fortified foods and dietary supplements to help achieve nutrient adequacy [1].

The consumption of dietary supplements has been shown to increase overall nutrient intake and decrease the prevalence of nutrient inadequacy [3,4]. However, the key consumer motivators for dietary supplement use are maintenance or improvement in overall health as well as specific health benefits rather than filling nutritional gaps; thus, supplements are primarily considered a favorable health and lifestyle choice [9,10]. The consumption of dietary supplements has increased over time in the United States [11] and currently about 50% adults take dietary supplements and more than two-thirds of these use vitamin/mineral supplements [12,13,14]. The high prevalence of dietary supplement use in the United States has increased interest in research evaluating the role of supplements in meeting nutritional requirements.

The primary objective of this cross-sectional study was to investigate age-related differences in effect of dietary supplements on nutrient intake and prevalence of inadequacy using a large nationally representative data set. This study was part of a broader effort to determine the effect of dietary supplements among diverse US populations.

## 2. Materials and Methods

### 2.1. Study Population

The National Health and Nutrition Examination Survey (NHANES) is a continuous nationally representative, cross-sectional survey of non-institutionalized, civilian U.S. residents and the data are collected by the National Center of Health Statistics (NCHS) of the Centers for Disease Prevention and Control and released in two year cycles. Data from NHANES 2009–2010 and 2011–2012 were combined for the present analyses, and included 10,698 adults (age 19 years and older), excluding pregnant or lactating females and those with incomplete dietary records or missing data [15]. All participants or proxies provided written informed consent and the Research Ethics Review Board at the NCHS approved the survey protocol. Participants were categorized into age groups: age 19–50 years (*n* = 5793), 51–70 years (*n* = 3330) and ≥71 years (*n* = 1575).

### 2.2. Micronutrient Intake from Foods

Dietary intake from foods was estimated from two reliable 24-h dietary recall interviews using United States Department of Agriculture’s (USDA) automated multiple-pass method (AMPM) [15]. The nutrient content of foods consumed by NHANES 2009–2010 and 2011–2012 participants was determined by using Food and Nutrient Database for Dietary Studies (FNDDS) 2009–2010, and 2011–2012 [16,17] in conjunction with USDA National Nutrient Database for Standard Reference (SR) releases 24 and 26 [18], respectively.

### 2.3. Micronutrient Intake from Supplements

A dietary supplement questionnaire assessing the usage of vitamins, minerals, botanicals, and other dietary supplements was administered as part of the NHANES household interview, and the consumption frequency, duration and dosage were recorded for each supplement used over the past 30 days [19]. The complete product information including labeled dosage or serving size, ingredients, and the amounts of ingredients per serving, was also recorded. The average daily intake of nutrients from dietary supplements was calculated using the supplement consumption frequency and dosage (i.e., the sum of all supplements taken calculated by the number of days taken in last 30 days times the amount of nutrient in each supplement taken divided by 30 days). In this way, the number of dietary supplements taken and the frequency of consumption of each dietary supplements taken was considered.

### 2.4. Statistics

All statistical analyses were performed with SAS software (version 9.2; SAS Institute Inc., Cary, NC, USA) and SUDAAN (version 11; Research Triangle Institute; Raleigh, NC, USA). Usual nutrient intakes (long-term intakes) from food only and from food plus dietary supplement for the entire population group were estimated using two days of dietary intake in NHANES with the National Cancer Institute method [20] and with day of recall, weekday/weekend intake flag, and dietary supplement use (yes/no) flag as covariates. Percentage of the population below the Estimated Average Requirement (EAR) using the cut-point method (except for iron where the probability method was used) for 17 nutrients (calcium, copper, iron, magnesium, phosphorus, selenium, zinc, vitamin A, thiamin, riboflavin, niacin, folate, vitamin B6, vitamin B12, vitamin C, vitamin D, and vitamin E), above the Adequate Intake (AI) for two nutrients (vitamin K and choline; given an EAR has not been established the percentage of the population with inadequate intakes cannot be determined [21]), and above the Upper Tolerable Level (UL) for 13 nutrients (calcium, copper, iron, phosphorus, selenium, zinc, vitamin A as retinol, folate as folic acid, vitamin B6, vitamin C, vitamin D, vitamin E as added alpha-tocopherol and choline) were assessed. EAR, AI, and UL used were age/gender specific. Potassium and sodium were excluded from the present analysis as negligible amounts are found in dietary supplements. To obtain nationally representative estimates, NHANES survey weights, strata, and primary sampling units were used in all calculations. A Z-statistic was used to test whether usual intake means and proportions of the population below EAR or above the UL were similar between groups. *p* < 0.01 was deemed significant. Data are presented as mean ± SE.

### 2.5. Trial Registration 

Not applicable, as this is secondary analysis of publicly released observational data (NHANES 2009–2012).

## 3. Results

### 3.1. Dietary Supplement Usage

Among NHANES 2009–2012 participants, dietary supplement use (mean ± standard error) was reported to be 45.8 ± 1.2% of adults age 19–50 years, 64.9 ± 1.3% of adults age 51–70 years, and 73.2 ± 1.1% of adults age ≥71 years.

### 3.2. Comparison of Intakes from Food Alone in Dietary Supplement Consumers and Non-Consumers

In adults 19–50 years, consumers of dietary supplements had higher (*p* < 0.01) intakes from food alone of most nutrients examined (not selenium, zinc and vitamin B_12_) as compared to non-consumers (Table 1). Consumers of dietary supplements 51–70 years had higher intakes of vitamins A, C and E and lower intake of choline while consumers ≥71 years had higher intakes of copper, magnesium, vitamins B_6_ and K as compared to non-consumers. Regarding the percentage of the population with inadequate intakes from food alone (Table 2), consumers of dietary supplements had lower inadequacy for magnesium (all three age groups), vitamin A (19–50 and 51–70 year groups), vitamin C (19–50 and 51–70 year groups), and copper (≥71 year group); the percentage of the population above the AI for vitamin K as higher for dietary supplement consumers for all three age groups. Both dietary supplement consumers and non-consumers had high percentages of the population below the EAR from food only for calcium, magnesium, and vitamins A, C, D, and E along with relatively low percentages of the population with intakes above the AI for vitamin K and choline.

### 3.3. Effect of Supplement Use on Usual Intake of Nutrients

Usual intake of nutrients from food and supplement combined was higher (*p* < 0.01) than from food only for all nutrients except phosphorus, vitamin K and choline (Table 3). Usual intakes of DGA-identified “underconsumed nutrients” increased (range of mean intake differences across age groups) significantly in the various age subgroups by 9–34% for calcium, 17–29% for iron, 5–16% for magnesium, 37–88% for vitamin A, 66–159% for vitamin C, 118–309% for vitamin D and 123–461% for vitamin E. The magnitude of increase was generally higher for older than for younger age subgroups (≥71 years > 51–70 years > 19–50 years) (Table 3), consistent with increased dietary supplement use in older age groups.

### 3.4. Effect of Supplement Use on Prevalence of Inadequacy

Consumption of dietary supplements decreased (*p* < 0.01) the prevalence of inadequacy (range of mean differences in intakes below EAR across age groups) for DGA-identified “underconsumed nutrients” by 18–35% for calcium, 10–19% for magnesium, 19–38% for vitamin A, 21–43% for vitamin C, 23–53% for vitamin D and 24–43% for vitamin E across all age groups, with the magnitude of decrease generally higher for older population subgroup (Table 4). There was also a decrease (*p* < 0.01) in the prevalence of intakes below the EAR for zinc (age ≥71 years), vitamin B6 (age ≥71 years and 51–70 years), and folate (age ≥71 years and 51–70 years) associated with dietary supplement intake. There were no significant differences in the prevalence of intakes above the AI of vitamin K or choline with supplement intake (plus food) compared to food alone among adults of any age group except the ≥71 years group had increased percentage of the population above the AI for vitamin K intakes with supplement intake.

### 3.5. Comparison of Prevalence of Inadequacy by Age

There were significant differences in proportion of population with intakes (from food + supplement) below EAR by age groups (Table 4). The ≥71 years population subgroup had a lower prevalence of inadequacy for iron, vitamin A, vitamin C, vitamin D, and vitamin E, and higher prevalence of inadequacy for calcium, magnesium and other minerals (except iron), and most B vitamins compared to other age subgroups. Among DGA-identified “underconsumed nutrients”, the ≥71 years subgroup, compared to the other two age subgroups, had (range of differences in mean intakes below EAR changes across age groups) 38–110% higher prevalence for calcium inadequacy, 98% lower prevalence for iron inadequacy (compared to age 19–50 years only), 28–32% higher prevalence for magnesium inadequacy, 18–40% lower prevalence for vitamin A inadequacy, 28% lower prevalence for vitamin C inadequacy (compared to age 19–50 years only), 17–40% lower prevalence for vitamin D inadequacy and 19% lower prevalence for vitamin E inadequacy (compared to age 19–50 years only).

### 3.6. Effect of Supplement Use on Prevalence of Intake Higher Than the UL

There was a higher (*p* < 0.01) prevalence of intakes above UL for calcium (all age groups), iron (all age groups), selenium (age 19–50 years and 51–70 years population subgroups), zinc (all age groups), vitamin A (all age groups), folate (all age groups), vitamin B6 (all age groups), vitamin C (age 19–50 years only), and vitamin D (all age groups) with dietary supplement consumption (Table 5). The actual percentages above the UL were mostly below 2% and were never more than 5% for any nutrient and any age group except for calcium (9.0%, age 51–70 years) and zinc (5.2%, age ≥71 years).

## 4. Discussion

This is among the first analysis of NHANES data to examine micronutrient intakes from food alone and food plus dietary supplements among different US population age subgroups. While there were some differences in intakes from food alone in dietary supplement consumers and non-consumers, especially in the 19–50 years group, all age groups had greater than 25% of the population with intakes below the EAR for calcium, magnesium, and vitamins A, C, D, and E. This analysis, based on NHANES data from 2009–2012, shows that dietary supplement use consistently contributed to increased intakes of nutrients and decreased population prevalence of inadequacy among all age subgroups with only small (typically < 5%) increases in the population exceeding the UL.

Inadequacy of nutrient intakes among Americans have been consistently demonstrated in past reports [1,3,4,5,6]. Our data show that older adults had lower intake and higher prevalence of inadequacy for most nutrients (from food only) compared to younger adults. Previous studies have also reported lower intake of several key nutrients among older adults. One study estimated that older adults consume only 20–33% of the Recommended Dietary Allowance for many nutrients [22]. A 2005–2006 NHANES analysis indicated that over 92% adults age 51+ were consuming below the EAR for vitamin E and 67% below the EAR for magnesium [23]. While good nutritional status in older adults can have beneficial effects on quality of life, morbidity, and mortality, this population appears at a disproportionately greater risk of inadequate intakes. Nutrient inadequacies were shown to be more pronounced in older adults, possibly due to a decrease in total energy intake with advancing age [24]. There is consistent data from both cohort and cross-sectional surveys indicating a substantial age-associated decline in energy intake and food intake quantity [22]. Additionally, a high proportion of older adults (age >71 years) had inadequate intakes for fruits (~70%), vegetables (>80%), and whole grains (>90%) in an earlier study [2].

Our analysis shows that use of dietary supplements significantly increased nutrient intakes and decreased the prevalence of inadequacy for most nutrients in all age subgroups. Calcium, potassium, iron (adolescent and adult females), magnesium, dietary fiber, choline, and vitamins A, D, E, and C are “underconsumed nutrients” as their intakes for many individuals are below the recommendations [1]. Increased risk of several adverse health effects including cardiovascular disease, stroke, impaired cognitive function, cancer, eye diseases, poor bone health and other conditions have been associated with nutrient inadequacies [8,25,26]. Low intakes of calcium, potassium, iron (adolescent and adult females) dietary fiber, and vitamin D are associated with the risk of chronic disease [1].

Although the prevalence of inadequacy for most nutrients from food only was higher among older adults, the prevalence of inadequacy (for intakes from food + supplements) for vitamins A, C, D and E among older adults was much lower than among younger adults. Dietary supplement use was associated with a lower prevalence of inadequacy in all age groups. The prevalence of inadequate intake of vitamins A, E, and folate among the elderly population decreased from 53% to 4%, 93% to 14%, and 75% to 7%, respectively, with the use of supplements [27]. It is interesting to note that, while there was generally an increased intake associated with dietary supplements across all age groups, the decrease in prevalence of inadequacy among older adults was much higher than among younger age groups. Our data show higher prevalence of dietary supplement use among older adults than among younger adults. Similar findings of higher incidence of dietary supplement use among older population subgroup were also reported in earlier studies. In the National Health Interview Survey (NHIS), while 53% of participants reported taking vitamin or mineral supplements, 60–61% of older Americans reported taking supplement [28]. More prevalent dietary supplement use among older adults was also reported from an NHANES 2003–2006 analysis [9] as well as from NHANES III [29]. It appears that although the older adult population subgroups typically start with lower intakes, their more prevalent use of dietary supplements help compensate for nutritional inadequacies.

A major strength of our study was the use of a large nationally representative population-based sample of adults. Our analysis relied on the assumption that 24-h dietary recall-based nutrient intakes from food sources were unbiased and self-reported dietary supplement intake accurately reflected long-term intake patterns. We assumed self-reported data for dietary supplement use from the NHANES questionnaire represents long-term supplement intake but this has not been validated. However, the NHANES interviewers used standardized procedures and saw the dietary supplement bottles/labels and verified the self-reporting accuracy 85% of the time [9]. Additionally, the estimates of vitamins and minerals contributed by dietary supplements relied on the label declarations rather than analytic values. The results of this study should be interpreted with these limitations in mind.

In conclusions, the results of this study suggest an association between supplement use and meeting nutritional adequacies for various nutrients, and that the association is stronger for older adult population subgroups than younger population ones.

## Figures and Tables

**Table 1:** Usual intake of nutrients from foods only among adults (19+ years old) by age groups. NHANES 2009–2012, gender combined data.

Nutrients	Age 19–50 Years		Age 51–70 Years		Age ≥71 Years	
	Non-Consumer (*n* = 3427)	Consumer (*n* = 2366)	Non-Consumer (*n* = 1408)	Consumer (*n* = 1922)	Non-Consumer (*n* = 490)	Consumer (*n* = 1085)
Nutrients with EAR (Estimated Average Requirement)						
Calcium (mg)	1026 ± 11	1109 ± 20 *	928 ± 18	968 ± 17	818 ± 24	861 ± 14
Copper (mg)	1.27 ± 0.02	1.42 ± 0.02 *	1.27 ± 0.04	1.39 ± 0.03	1.09 ± 0.03	1.20 ± 0.02 *
Iron (mg)	15.6 ± 0.1	16.6 ± 0.2 *	14.9 ± 0.4	15.2 ± 0.3	14.2 ± 0.5	14.5 ± 0.3
Magnesium (mg)	303 ± 4	338 ± 5 *	300 ± 7	318 ± 5	249 ± 7	274 ± 4 *
Phosphorus (mg)	1470 ± 12	1538 ± 21 *	1362 ± 22	1358 ± 18	1135 ± 26	1183 ± 18
Selenium (µg)	120 ± 1	122 ± 12	112 ± 2	108 ± 2	92.4 ± 3.3	91.9 ± 1.2
Zinc (mg)	12.1 ± 0.1	12.6 ± 0.2	11.3 ± 0.2	11.3 ± 0.2	10.0 ± 0.2	10.4 ± 0.2
Vitamin A (µg RE)	568 ± 12	683 ± 17 *	604 ± 21	749 ± 43 *	639 ± 26	676 ± 22
Thiamin (mg)	1.67 ± 0.02	1.80 ± 0.03 *	1.59 ± 0.03	1.60 ± 0.03	1.46 ± 0.04	1.46 ± 0.2
Riboflavin (mg)	2.09 ± 0.03	2.34 ± 0.04 *	2.14 ± 0.04	2.19 ± 0.05	1.93 ± 0.06	1.99 ± 0.03
Niacin (mg)	27.9 ± 0.43	28.1 ± 0.4 *	25.0 ± 0.6	24.2 ± 05	20.6 ± 0.5	21.3 ± 0.3
Folate (µg DFE)	567 ± 8	600 ± 11 *	519 ± 15	555 ± 16	490 ± 19	507 ± 11
Vitamin B_6_ (mg)	2.19 ± 0.04	2.34 ± 0.05 *	2.07 ± 0.07	2.01 ± 0.05	1.74 ± 0.04	1.90 ± 0.04 *
Vitamin B_12_ (µg)	5.33 ± 0.09	5.73 ± 0.03	5.15 ± 0.15	5.18 ± 0.25	4.70 ± 0.17	4.98 ± 0.18
Vitamin C (mg)	79.8 ± 2.5	89.4 ± 2.7 *	78.4 ± 4.1	95.2 ± 1.9 *	73.2 ± 4.4	85.9 ± 3.7
Vitamin D (µg)	4.66 ± 0.10	5.05 ± 0.17	4.84 ± 0.13	4.83 ± 0.17	5.10 ± 0.25	5.03 ± 0.18
Vitamin E (mg)	8.25 ± 0.17	9.2 ± 0.2 *	7.81 ± 0.27	9.02 ± 0.21 *	6.80 ± 0.33	7.56 ± 0.16
Nutrients with AI (Adequate Intake)						
Vitamin K (µg)	90.1 ± 2.3	111 ± 5 *	108 ± 7	131 ± 8	79.7 ± 5.5	106 ± 5 *
Choline (mg)	344 ± 5	354 ± 5	351 ± 6	329 ± 5 *	287 ± 9	288 ± 5

* Significant difference for consumer and non-consumer within age subgroups at *p* < 0.01.

**Table 2:** Percent of adult (19+ years old) population below Estimated Average Requirement (EAR) or above Adequate Intake (AI) of nutrients from foods only. NHANES 2009–2012 gender combined data.

Nutrients	Age 19–50 Years		Age 51–70 Years		Age ≥71 Years	
	Non-Consumer (*n* = 3427)	Consumer (*n* = 2366)	Non-Consumer (*n* = 1408)	Consumer (*n* = 1922)	Non-Consumer (*n* = 490)	Consumer (*n* = 1085)
Nutrients with EAR, percentage below EAR						
Calcium	29.5 ± 1.4	25.4 ± 1.4	53.7 ± 1.9	50.7 ± 1.9	75.0 ± 2.9	71.8 ± 19
Copper	6.0 ± 0.7	2.8 ± 0.8	7.8 ± 1.1	2.2 ± 0.6 *	15.0 ± 3.0	7.2 ± 1.2
Iron	6.6 ± 0.7	5.9 ± 0.6	<1	<1	<1	<1
Magnesium	55.0 ± 1.7	40.1 ± 1.4 *	59.8 ± 2.6	46.9 ± 1.8 *	77.8 ± 3.4	65.1 ± 2.1 *
Phosphorus	<1	<1	<1	<1	4.4 ± 1.2	1.5 ± 0.5
Selenium	<1	<1	<1	<1	3.3 ± 1.6	2.1 ± 0.8
Zinc	11.9 ± 1.6	8.2 ± 1.8	20.3 ± 2.0	16.7 ± 1.6	31.8 ± 3.2	23.8 ± 2.1
Vitamin A	55.0 ± 2.3	39.5 ± 2.2 *	51.7 ± 2.8	31.9 ± 3.9 *	45.5 ± 4.2	33.8 ± 3.4
Thiamin	4.5 ± 1.3	3.7 ± 0.8	5.3 ± 1.3	6.3 ± 1.2	13.2 ± 2.7	7.3 ± 1.8
Riboflavin	3.2 ± 0.7	1.5 ± 0.5	3.7 ± 0.7	2.0 ± 0.5	5.7 ± 1.6	2.7 ± 0.5
Niacin	<1	<1	<1	1.6 ± 0.4	6.2 ± 2.0	3.1 ± 0.9
Folate DFE	8.3 ± 2.3	8.2 ± 1.3	13.0 ± 1.8	9.0 ± 1.6	20.6 ± 3.4	15.9 ± 1.8
Vitamin B_6_	4.6 ± 1.1	3.2 ± 1.0	15.0 ± 2.6	16.4 ± 1.7	27.3 ± 2.2	20.4 ± 2.0
Vitamin B_12_	2.0 ± 0.8	2.5 ± 1.0	6.3 ± 1.7	4.5 ± 1.4	5.5 ± 1.9	5.0 ± 1.5
Vitamin C	49.1 ± 2.0	39.2 ± 2.1 *	52.3 ± 2.7	36.2 ± 2.0 *	53.1 ± 3.9	40.9 ± 2.7
Vitamin D	96.8 ± 0.8	92.8 ± 1.4	93.8 ± 1.1	94.9 ± 1.4	94.4 ± 2.1	96.0 ± 1.2
Vitamin E	86.5 ± 1.6	80.7 ± 1.5	90.3 ± 2.4	81.9 ± 1.9	93.6 ± 2.3	91.2 ± 1.3
Nutrients with AI, percentage above AI						
Vitamin K	29.3 ± 2.2	43.9 ± 3.0 *	40.3 ± 4.0	57.4 ± 3.1 *	23.7 ± 3.9	42.5 ± 3.1 *
Choline	8.4 ± 1.2	10.3 ± 1.3	9.9 ± 1.6	6.8 ± 0.9	2.3 ± 0.9	2.0 ± 0.8

* Significant difference for consumer and non-consumer within age subgroups at *p* < 0.01.

**Table 3:** Usual intake of nutrients from foods and foods + dietary supplements among adults (19+ years old) by age groups. NHANES 2009–2012, gender combined data.

Nutrients	Age 19–50 Years (*n* = 5793)		Age 51–70 Years (*n* = 3330)		Age ≥71 Years (*n* = 1575)	
	Food Only	Food + Supplement	Food Only	Food + Supplement	Food Only	Food + Supplement
Nutrients with EAR						
Calcium (mg)	1065 ± 11	1157 ± 13 *	956 ± 20	1192 ± 26 *	848 ± 11	1135 ± 15 *
Copper (mg)	1.34 ± 0.02	1.60 ± 0.02 *	1.35 ± 0.02	1.68 ± 0.03 *	1.17 ± 0.02	1.60 ± 0.04 *
Iron (mg)	16.1 ± 0.1	18.7 ± 0.2 *	15.1 ± 0.2	18.2 ± 0.3 *	14.4 ± 0.3	18.6 ± 0.4 *
Magnesium (mg)	319 ± 3	337 ± 3 *	312 ± 4	351 ± 6 *	267 ± 3	311 ± 5 *
Phosphorus (mg)	1501 ± 12	1506 ± 11	1358 ± 18	1369 ± 19	1169 ± 14	1184 ± 13
Selenium (µg)	121 ± 1	133 ± 2 *	110 ± 2	137 ± 7 *	91.9 ± 1.4	118 ± 2 *
Zinc (mg)	12.3 ± 0.1	15.2 ± 0.2 *	11.3 ± 0.2	16.1 ± 0.4 *	10.3 ± 0.2	17.8 ± 0.5 *
Vitamin A (µg RE)	622 ± 12	853 ± 13 *	700 ± 29	1099 ± 46 *	666 ± 18	1254 ± 47 *
Thiamin (mg)	1.73 ± 0.02	4.19 ± 0.18 *	1.59 ± 0.02	7.06 ± 0.85 *	1.46 ± 0.02	7.50 ± 2.40
Riboflavin (mg)	2.20 ± 0.02	4.13 ± 0.13 *	2.18 ± 0.05	5.88 ± 0.79 *	1.97 ± 0.03	4.80 ± 0.57 *
Niacin (mg)	28.0 ± 0.3	34.5 ± 0.7 *	24.5 ± 0.3	38.6 ± 1.8 *	21.2 ± 0.3	33.0 ± 1.2 *
Folate (µg DFE)	582 ± 6	740 ± 7 *	543 ± 8	798 ± 15 *	503 ± 10	820 ± 15 *
Vitamin B_6_ (mg)	2.26 ± 0.03	4.70 ± 0.13 *	2.03 ± 0.03	6.38 ± 0.48 *	1.87 ± 0.03	6.44 ± 0.55 *
Vitamin B_12_ (µg)	5.51 ± 0.10	32.4 ± 2.9 *	5.18 ± 0.15	69.8 ± 7.4 *	4.91 ± 0.14	101 ± 10 *
Vitamin C (mg)	84.6 ± 2.0	141 ± 5 *	89.5 ± 2.0	192 ± 11 *	82.5 ± 2.4	214 ± 14 *
Vitamin D (µg)	4.83 ± 0.11	10.6 ± 0.7 *	4.83 ± 0.12	18.1 ± 1.2	5.06 ± 0.17	20.7 ± 1.4 *
Vitamin E (mg)	8.69 ± 0.11	19.4 ± 0.9 *	8.59 ± 0.13	32.8 ± 2.2 *	7.35 ± 0.16	41.2 ± 3.1 *
Nutrients with AI						
Vitamin K (µg)	99.6 ± 2.4	105 ± 2	124 ± 4	132 ± 4	99.5 ± 3.3	110 ± 3
Choline (mg)	348 ± 3	349 ± 3	337 ± 5	340 ± 5	287 ± 5	289 ± 5

* Significantly different from Food Only at *p* < 0.01.

**Table 4:** Percent of adult (19+ years old) population below Estimated Average Requirement (EAR) or above Adequate Intake (AI) of nutrients from foods and foods + dietary supplements by age groups. NHANES 2009–2012 gender combined data.

Nutrients	Age 19–50 Years (*n* = 5793)		Age 51–70 Years (*n* = 3330)		Age ≥71 Years (*n* = 1575)	
	Food Only	Food + Supplement	Food Only	Food + Supplement	Food Only	Food + Supplement
Nutrients with EAR, percentage below EAR						
Calcium	27.5 ± 0.9	22.7 ± 0.9 *^,a^	51.4 ± 1.5	34.6 ± 1.2 *^,b^	72.9 ± 1.6	47.7 ± 1.2 *^,c^
Copper	4.6 ± 0.5	4.1 ± 0.5 ^a^	4.1 ± 0.8	3.1 ± 0.6 ^a^	9.6 ± 1.3	6.7 ± 0.7 ^b^
Iron	6.2 ± 0.5	5.0 ± 0.4 ^a^	<1	<1 ^b^	<1	<1 ^b^
Magnesium	47.8 ± 1.2	43.0 ± 1.1 *^,a^	51.3 ± 1.5	41.9 ± 1.2 *^,a^	68.6 ± 1.5	55.2 ± 1.5 *^,b^
Phosphorus	<1	<1 ^a^	<1	<1 ^a,b^	2.07 ± 0.51	2.0 ± 0.4 ^b^
Selenium	<1	<1 ^a^	<1	<1 ^a^	2.4 ± 0.7	1.5 ± 0.4 ^b^
Zinc	10.1 ± 1.5	8.08 ± 1.25 ^a^	17.9 ± 1.9	12.6 ± 1.0 ^b^	26.1 ± 1.8	16.4 ± 1.0 *^,c^
Vitamin A	47.3 ± 1.7	38.2 ± 1.2 *^,a^	39.2 ± 2.2	28.2 ± 1.6 *^,b^	37.4 ± 2.9	23.0 ± 1.6 *^,b^
Thiamin	4.2 ± 0.8	3.5 ± 0.7	6.0 ± 1.0	4.1 ± 0.5	8.9 ± 1.8	5.3 ± 0.9
Riboflavin	2.5 ± 0.5	2.10 ± 0.42	2.6 ± 0.4	1.9 ± 0.3	3.4 ± 0.5	2.2 ± 0.3
Niacin	<1	<1 ^a^	1.3 ± 0.4	<1 ^a^	4.0 ± 0.9	2.5 ± 0.5 ^b^
Folate DFE	8.3 ± 1.2	6.4 ± 0.9 ^a^	10.6 ± 1.0	7.4 ± 0.7 *^,a,b^	17.0 ± 1.7	10.3 ± 1.0 *^,b^
Vitamin B_6_	4.0 ± 0.7	3.2 ± 0.5 ^a^	15.6 ± 1.3	10.5 ± 0.8 *^,b^	22.4 ± 1.8	14.0 ± 1.1 *^,c^
Vitamin B_12_	2.3 ± 0.6	1.7 ± 0.5	5.2 ± 1.4	3.3 ± 0.9	4.9 ± 1.1	2.8 ± 0.5
Vitamin C	44.6 ± 1.4	35.2 ± 1.2 *^,a^	42.1 ± 1.9	28.7 ± 1.2 *^,b^	44.2 ± 1.9	25.4 ± 1.3 *^,b^
Vitamin D	94.8 ± 0.9	73.5 ± 1.0 *^,a^	94.6 ± 0.9	53.3 ± 1.2 *^,b^	95.5 ± 1.2	44.4 ± 1.3 *^,c^
Vitamin E	83.8 ± 0.9	64.1 ± 0.8 *^,a^	85.0 ± 1.3	55.3 ± 1.0 *^,b^	91.7 ± 1.2	52.1 ± 1.4 *^,b^
Nutrients with AI, percentage above AI						
Vitamin K	37.2 ± 1.7	41.3 ± 1.7 ^a^	51.3 ± 2.0	56.6 ± 2.2 ^b^	37.1 ± 1.8	45.1 ± 1.5 *^,a^
Choline	8.9 ± 0.8	9.2 ± 0.8 ^a^	7.8 ± 0.9	8.3 ± 1.1 ^a^	2.1 ± 0.6	2.2 ± 0.5 ^b^

* Significantly different from Food Only at *p* < 0.01; ^a,b,c^ Values with different letters in a row are significantly different at *p* < 0.01.

**Table 5:** Percent adult (19+ years old) population exceeding Tolerable Upper Limit of intake (UL) of nutrients from foods and foods + dietary supplements by age groups. NHANES 2009–2012 gender combined data.

Nutrients	Age 19–50 Years (*n* = 5793)		Age 51–70 Years (*n* = 3330)		Age ≥71 Years (*n* = 1575)	
	Food Only	Food + Supplement	Food Only	Food + Supplement	Food Only	Food + Supplement
Calcium	<1	1.24 ± 0.20 *	1.45 ± 0.43	9.00 ± 1.08 *	<1	<1
Copper	<1	<1	<1	<1	<1	<1
Iron	<1	1.37 ± 0.17 *	<1	1.98 ± 0.29 *	<1	2.63 ± 0.39 *
Phosphorus	<1	<1	<1	<1	<1	<1
Selenium	<1	<1	<1	<1	<1	<1
Zinc	<1	1.10 ± 0.17 *	<1	2.53 ± 0.41 *	<1	5.20 ± 0.62 *
Vitamin A	<1	<1	<1	<1	<1	<1
Niacin	ND	ND	ND	ND	ND	ND
Folate DFE	<1	1.35 ± 0.13 *	<1	1.96 ± 0.30 *	<1	2.63 ± 0.40 *
Vitamin B_6_	<1	<1	<1	1.42 ± 0.38 *	<1	1.95 ± 0.39 *
Vitamin C	<1	<1	<1	<1	<1	<1
Vitamin D	<1	<1	<1	1.95 ± 0.67 *	<1	1.99 ± 0.57 *
Vitamin E	<1	<1	<1	<1	<1	<1
Choline	<1	<1	<1	<1	<1	<1

* Significantly different from Food Only at *p* < 0.01; Vitamin A, folate and vitamin E ULs based on retinol, folic acid and added alpha tocopherol, respectively. ND: Not determined as niacin UL is based on a particular form of niacin (nicotinic acid) which is not quantified in NHANES.
